# Infant- and Young Child-feeding Practices in Bankura District, West Bengal, India

**DOI:** 10.3329/jhpn.v28i3.5559

**Published:** 2010-06

**Authors:** Apurba Sinhababu, Dipta K. Mukhopadhyay, Tanmay K. Panja, Asit B. Saren, Nirmal K. Mandal, Akhil B. Biswas

**Affiliations:** Department of Community Medicine, B.S. Medical College, Bankura 722 102, West Bengal, India

**Keywords:** Breastfeeding, Child-feeding practices, Cross-sectional studies, Descriptive studies, Exclusive breastfeeding, Infant-feeding practices, India

## Abstract

A community-based, cross-sectional descriptive study was conducted during June-July 2008 to assess the infant- and young child-feeding (IYCF) practices in Bankura district, West Bengal, India. In total, 647 children aged less than two years selected through revised 40-cluster sampling using the indicators of the Integrated Management of Neonatal and Childhood Illness (IMNCI) and World Health Organization. The proportions of infants with early initiation of breastfeeding (13.6%) and exclusive breastfeeding under six months (57.1%) and infants who received complementary feeding at the age of 6–8 months (55.7%) were low. Appropriate feeding as per the IMNCI protocol was significantly less among infants aged 6–11 months (15.2%) and children aged 12–23 months (8.7%) compared to infants aged less than six months (57.1%), which could be attributable to low frequency and amount of complementary feeding. The main problems revealed from the study were late initiation of breastfeeding, low rates of exclusive breastfeeding, and inappropriate complementary feeding practices.

## INTRODUCTION

Optimal infant- and young child-feeding (IYCF) practices are crucial for nutritional status, growth, development, health, and ultimately the survival of infants and young children (1–3). Worldwide, suboptimal breastfeeding still accounts for deaths of 1.4 million children aged less than five years (under-five mortality). The timely introduction of complementary feeding can prevent almost 6% of under-five mortality (4). It was estimated that, if 90% of infants are covered with a package of intervention to protect, promote, and support the optimal IYCF practices, almost one-fifth of overall under-five mortality can be averted (4). The poor complementary feeding practices mean that many children continue to be vulnerable to irreversible outcomes of stunting, poor cognitive development, and significantly increased risk of infectious diseases, such as diarrhoea and acute respiratory infection (3, 5, 6).

This has a tremendous impact in a developing country, like India, with a high burden of disease and low access to safe water and sanitation. Even in developed countries, recent studies have underscored the role of IYCF practices in reducing child mortality (7, 8).

The World Health Organization (WHO) recommends exclusive breastfeeding for the first six months of life with early initiation and continuation of breastfeeding for two years or more together with nutritionally-adequate, safe, age-appropriate complementary feeding starting at six months (9). The WHO and United Nations Children Fund (UNICEF) have articulated a global strategy for infant- and young child-feeding. Based on these guiding principles, the Government of India, in collaboration with international agencies, has adopted the culturally-acceptable IYCF guidelines, which were incorporated in the Integrated Management of Neonatal and Childhood Illness (IMNCI) Programme (10).

The National Family Health Survey (NFHS-3) has provided useful national- and state-level information on the IYCF practices (11). Available data showed a gross inter-state variation. However, the NFHS was not designed to provide district-level data. With this background, the present study was undertaken to assess the IYCF practices among children aged less than two years in Bankura district, West Bengal, as part of the “Multi-indicator survey on delivery and child-caring practices in Bankura district, West Bengal”.

## MATERIALS AND METHODS

### Study setting, design, and sample

A community-based, cross-sectional descriptive study was conducted in Bankura district of West Bengal among children aged less than two years during June-July 2008 using the two-stage revised 40-cluster sampling technique (12).

In absence of any previous study in the district, the lowest figure of all the IYCF indicators as reported in the NFHS-3 (i.e. 24.5% for early initiation of breastfeeding) was used for calculating the sample size. Considering 95% confidence level, 20% relative precision, and design effect 2, the yielded sample size was 616. It was rounded off to 640 for an equal subsample of 16 from each of 40 clusters (villages/urban wards).

In the first stage, 40 clusters (villages/urban wards) were selected based on the probability proportional to size. Each selected cluster was divided into four arbitrary quadrants. With a random start, consecutive households were visited in each quadrant to select four children aged less than two years to include a total of 16 children per cluster. All the eligible children in the last household of each quadrant were included even if the number exceeded four.

Trained interviewers collected information on age, sex, and feeding practices of the study children by interviewing mothers/other responsible caregivers at their home. All feeding practices for children were elicited using the 24-hour recall method, except for initiation of breastfeeding and prelacteal feeding, for which historic recall was used. Definitions formulated by the WHO for indicators of the IYCF practices were used (13). However, for computing appropriate feeding, the IMNCI guidelines were followed (10) (Table 1).

**Table 1.** Age-specific ‘appropriate feeding’ as per the IMNCI guidelines

Age-group (months)	Breastfed	Type of food	Frequency and amount
0–5	Yes	Only breastmilk	8 times or more per 24 hours
6–11	Yes	Solid/semi-solid/soft food	Minimum 1 katori* 3 times
	No	Solid/semi-solid/soft food	Minimum1 *katori* 5 times
12–23	Irrespective of status	Solid/semi-solid/soft food	Minimum 1.5 *katori* 5 times

*Katori≈150 g;

IMNCI=Integrated Management of Neonatal and Childhood Illness

### Analysis of data

For consistency and inter-observer agreement, standardized proforma was used, and collected data were cross-checked and supervised. Analysis was done using the SPSS software (version 13.0), along with duplicate entry to check consistency of data. Simple proportions were calculated for each IYCF indicator. The differences in the feeding practices between sexes and age-groups, if any, were noted using chi-square test.

### Ethics

The Institutional Ethics Committee of the B.S. Medical College, Bankura, approved the study.

## RESULTS

In total, 3,318 households in 40 clusters were covered during the survey in Bankura district. The total number of people covered in the survey was 18,136; the average family size was 5.5+2.7. Children aged less than five and two years comprised 9.6% and 4.2% of the surveyed population respectively.

Although breastfeeding was universal, only 13.6% of the study children were put to the breast within one hour of birth. About 5% of the neonates (n=647) had to wait for at least 24 hours for first sips of breastmilk. The difference between age-groups in the proportion of children having early initiation of breastfeeding, although not significant, is shown in Table 2. Sex was not associated with early initiation of breastfeeding. The overall prevalence of prelacteal feeding was 26.7%, which showed a very little variation among age-groups or sexes. The major types of prelacteal feeds were sweetened water, animal-milk, especially goat's milk, and honey.

**Table 2.** Initial breastfeeding practices of study children according to age and sex

Age (months) and sex	Total	Early initiation*	χ^2^ (p value)	Prelacteal feeding†	χ^2^ (p value)
0–5					
Male	67	15 (22.4)	3.32	19 (28.4)	0.05
Female	73	8 (11.0)	(0.068)	22 (30.1)	(0.817)
Total	140	23 (16.4)*	df=1	41 (29.3)†	df=1
6–11					
Male	84	9 (10.7)	1.09	18 (21.4)	1.74
Female	100	16 (16.0)	(0.297)	30 (30.0)	(0.187)
Total	184	25 (13.6)*	df=1	48 (26.1)†	df=1
12–23					
Male	150	19 (12.7)	0.02	42 (28.0)	0.58
Female	173	21 (12.1)	(0.886)	42 (24.3)	(0.447)
Total	323	40 (12.4)*	df=1	84 (26.0)†	df=1
0–23					
Male	301	43 (14.3)	0.22	79 (26.2)	0.07
Female	346	45 (13.0)	(0.630)	94 (27.2)	(0.792)
Total	647	88 (13.6)	df=1	173 (26.7)	df=1

*χ^2^=1.36, df=2, p=0.507;

†χ^2^=0.59, df=2, p=0.7438;

Figures in parentheses in Column 3 and 5 indicate percentages;

df=Degree of freedom

As per 24-hour recall, 57.1% of the infants aged less than six months were exclusively breastfed. The exclusive breastfeeding rate dropped from 75.0% in infants aged less than two months to 48.7% at 2–3 months and 39.0% at 4–5 months of age. Similarly, the proportion of infants who were given only plain water, along with breastfeeding, decreased with age up to six months, and the overall prevalence was 14.3% at the age-group of 0–5 months (Table 3).

**Table 3.** Current feeding status of study children by age

Age (months)	Not breastfeeding	Exclusive breastfeeding	Breastfeeding and consuming			Total	Appropriate feeding
			Plain water	Other milk	Complementary Feeding		
<2	1 (1.7)	45 (75.0)	10 (16.7)	4 (6.7)	0 (0.0)	60 (100)	45 (75.0)
2–3	2 (5.1)	19 (48.7)	6 (15.4)	12 (30.8)	0 (0.0)	39 (100)	19 (48.7)
4–5	3 (7.3)	16 (39.0)	4 (9.8)	14 (34.2)	4 (9.8)	41 (100)	16 (39.0)
0–5	6 (4.3)	80 (57.1)	20 (14.3)	30 (21.4)	4 (2.9)	140(100)	80 (57.1)
6–8	4 (3.8)	5 (4.7)	0 (0.0)	38 (35.9)	59 (55.7)	106 (100)	15 (14.2)
9–11	1 (1.3)	0 (0.0)	0 (0.0)	4 (5.1)	73 (93.6)	78 (100)	13 (16.7)
12–23	29 (8.9)	0 (0.0)	0 (0.0)	7 (2.2)	287 (88.8)	323 (100)	28 (8.7)

Figures in parentheses indicate percentages

Conversely, the consumption of ‘other milk’ (formula milk and animal-milk), in addition to breastfeeding, was 6.7% in infants aged less than two months and increased to around 30% and more at 2–3 months onwards and declined sharply at 9–11 months of age (5.1%). The premature introduction of liquids other than breastmilk and soft/semi-solid food were found in 40.0% and 2.9% of the infants aged less than six months respectively (Table 3).

The continued breastfeeding rates at one year (12–15 months) and two years (20–23 months) were 97.9% and 88.6% respectively.

It was observed that 93.6% of all the breastfeeding infants aged less than six months were breastfed for eight times or more in the last 24 hours preceding the survey, the average number of feeds being 12.3 (+2.3). All the exclusively-breastfed infants aged 0–5 months received breastmilk eight times or more.

Fifty-nine (55.7%) infants aged 6–8 months were given semi-solid/solid food in addition to continued breastfeeding, and the proportion increased to 93.6% at the age of 9–11 months and declined thereafter (Table 3).

No infants aged less than two months used bottle-feeding. On the whole, 10.2% of the study children were bottle-fed.

In 0–5 months, adherence to ‘appropriate feeding’ was equal to exclusive breastfeeding, i.e. 57.1%. Table 2 shows that, in the age-group of 6–8 months and thereafter, huge disparity was observed between the proportion of children receiving complementary feeding and the proportion adhered to age-appropriate feeding as per the IMNCI guidelines. The overall proportion of appropriate feeding in children aged 0–23 months was 21.0%, with 15.2%% at 6–11 months of age and 8.7% at 12–23 months of age (Table 4).

**Table 4.** Age-specific appropriate feeding as per IMNCI protocol

Age (months)	Appropriate feeding as per IMNCI protocol			χ^2^ (p value)
	Yes	No	Total	
0–5	80 (57.1)*	60 (42.9)	140	0.86 (0.353) df=1
Male	41 (61.2)	26 (38.8)	67
Female	39 (53.4)	34 (46.6)	73
6–11	28 (15.2)*	156 (84.8)	184	1.76 (0.185) df=1
Male	16 (19.0)	68 (81.0)	84
Female	12 (12.0)	88 (88.0)	100
12–23	28 (8.7)*	295 (91.3)	323	0.63 (0.428) df=1
Male	15 (10.0)	135 (90.0)	150
Female	13 (7.5)	160 (92.5)	173
0–23	136 (21.0)	511 (79.0)	647	2.85 (0.091) df=1
Male	72 (23.9)	229 (76.1)	301
Female	64 (18.5)	282 (81.5)	346

*χ^2^=143.45; df=2; p<0.00001;

Figures in parentheses in Column 2 and 3 indicate percentages;

df=Degree of freedom;

IMNCI=Integrated Management of Neonatal and Childhood Illness

## DISCUSSION

Early initiation of breastfeeding, exclusive breastfeeding for six months, and timely introduction of age-appropriate complementary feeding are the key interventions to achieve the Millennium Development Goal 1 and 4, which address child malnutrition component of the targets and mortality respectively (1, 14).

The present study revealed that initiation of breastfeeding within one hour of birth was less common than the corresponding national (24.5%) and West Bengal (23.5%) figures of the NFHS 3 (11, 15) and far short of the 10^th^ plan target of 50% (16). A study in Ghana reported that 22% of all neonatal deaths could be prevented if all women could initiate breastfeeding within one hour of delivery (17). An epidemiological evidence of a causal association between early initiation of breastfeeding and infection-specific neonatal mortality has also been documented (18).

The use of prelacteal feeding was also far less compared to the corresponding NFHS 3 figures for India (57.2%) and West Bengal (47.8%). Similar figure was reported by Roy *et al.* (19). The corresponding figures were 8% in rural Bangladesh (3) and 71% in urban Bangladesh (20). Although this practice was prevalent across the cultures, there was an international consensus that providing other liquids in addition to breastmilk in the first six months of life was unnecessary and harmful (21).

Exclusive breastfeeding under six months (57.1%) in the present study was far short of the 10^th^ plan target of 80% (16), although better than all-India average of 46.4% (11) and similar to West Bengal (58.6%) (15). The declining rate of exclusive breastfeeding with age was also reported in the NFHS 3 study (11, 15) and could be attributed to supplementation with plain water in early months and milk other than breastmilk in later months. Similar findings were observed by Saha *et al.* in Bangladesh (3) and Hop *et al.* in Viet Nam (6) in longitudinal studies. Studies in India with historic recall noted that about one-fourth of study children received exclusive breastfeeding for six months (19, 24). Explanations put forward for this in different studies include beliefs that breastmilk does not contain water, and breastmilk alone is insufficient (25, 26). Several studies showed that partial breastfeeding was associated with increased risk of child morbidity and mortality (1, 2, 4). Even introduction of plain water was reported to interfere with breastfeeding (27). If the practice of giving plain water could be avoided, almost 15% increase in exclusive breastfeeding rate could be achieved. About one-fourth of study children who received liquids and solids, along with breastfeeding at 0–6 months of age, remained at risk for infectious diseases and undernutrition (2, 4).

Because of associated exposure to pathogens and interference with successful breastfeeding, current recommendations strongly discouraged bottle-feeding (28). The proportion of bottle-feeding in the present study was comparable with results of a study by Wamani *et al.* (25) but less than that reported by Pandey *et al.* from rural West Bengal (29).

Cousens *et al.* found that, when prolonged breastfeeding was accompanied with complementary solid foods, there was a reduction in clinical malnutrition (30). In the present study, the proportion of infants, aged 6–8 months, who received solid, semi-solid or soft food, in addition to breastmilk, was low (55.7%) when viewed against the 10^th^ plan target of 75% (16) but slightly better compared to India (52.7%) and West Bengal (47.1%) (11, 15). This low figure could be due to the increased use of ‘other types of milk’ till 6–8 months of age. It is worthwhile to note that the percentage of complementary feeding improved a lot at 9–11 months of age (93.6%). Saha *et al.* and Hop *et al.* have corroborated the findings (3, 6). A wide variation in the proportion of children who received complementary feeding at 6–9 months of age was reported from two other studies done in India (71.7% in Kolkata and 38.7% in Allahabad) (19, 24).

Studies in Malawi revealed that children who were given foods according to the time schedule recommended by the WHO were found to be well-nourished compared to children who received complementary feeding early (31). The current recommendations advocated the introduction of complementary food after six months of exclusive breastfeeding (9, 28). A positive association was observed between the intake of complementary food and the nutritional status in Yemen (32). A study in Bangladesh documented that the frequency, amount, energy-density, and diversity of food remained important issues in complementary feeding (33). The Indian guidelines on IYCF included minimum meal-frequency and amount per meal to compute age-appropriate feeding (10). Factors, such as characteristics of diet or child's appetite, are known to influence the frequency of complementary feeding (25, 26). Although these were not measured in this study, it is unlikely that such factors could solely explain the observed deviance from recommendations. Traditional beliefs and practices, besides lack of knowledge regarding current feeding recommendations, might also play a part (34, 35). The main problems that came out from the present study were late initiation of breastfeeding, low rates of exclusive breastfeeding, and inappropriate complementary feeding practices. Giving water and ‘milk other than breastmilk’ to breastfed babies were the limiting factors for exclusive breastfeeding. As for inappropriate complementary feeding practices, late introduction, low frequency, and inadequate amount of solid or semi-solid food turned out to be the areas of main thrust.

Area-specific programmes are to be planned to create an enabling environment for comprehensive nutrition and health education of mothers/care-givers, health and nutrition workers to protect, promote, and sustain the optimal IYCF practices in Bankura district.

## ACKNOWLEDGEMENTS

The authors gratefully acknowledge the financial support rendered by District Health and Family Welfare Samiti, Bankura and the all-round support extended by the teachers of the Department of Community Medicine, B.S. Medical College, Bankura.
